# Dehydrometabolites of siphonaxanthin, a carotenoid from green algae, suppress toll-like receptor 1/2-induced inflammatory response more strongly than siphonaxanthin

**DOI:** 10.1016/j.jbc.2025.108246

**Published:** 2025-01-31

**Authors:** Yuki Manabe, Tomoaki Nitta, Misato Ichihara, Takashi Maoka, Tatsuya Sugawara

**Affiliations:** 1 Division of Applied Biosciences, Graduate School of Agriculture, Kyoto University, Kyoto, Japan; 2 Division of Food Function and Chemistry, Research Institute for Production and Development, Kyoto, Japan

**Keywords:** carotenoid, inflammation, nuclear factor 2 (erythroid-derived 2-like factor), monocyte, toll-like receptor, interferon regulatory factor, metabolic conversion, stimulator of interferon genes

## Abstract

Siphonaxanthin (3,19,3′-trihydroxy-7,8-dihydro-**β**,ε-caroten-8-one) is a carotenoid found in green algae that exhibits potent anti-inflammatory activities. We previously reported that ingested siphonaxanthin accumulates in various organs of mice; however, its metabolic conversion remains largely unknown. In this study, we isolated three siphonaxanthin dehydrometabolites and determined their chemical structures. Two of these metabolites were obtained using the postmitochondrial supernatant prepared from mouse liver, whereas the third was obtained using the postmitochondrial supernatant prepared from rat liver. The human liver S9 fraction also generated two metabolites: one was identical to one of the rat metabolites, and the other was identical to one of the mouse metabolites. ^1^H-NMR revealed that all three metabolites had one or two additional **α**,**β**-unsaturated carbonyl groups (compared with siphonaxanthin). We also evaluated their anti-inflammatory activities and found that these three metabolites suppressed toll-like receptor 1/2–mediated interferon regulatory factor (IRF) activation more potently than siphonaxanthin. Pharmacological inhibition studies revealed that activation of nuclear factor erythroid 2–related factor 2 (Nrf2) is crucial for the inhibition of IRF activation by these metabolites. The Nrf2-mediated decrease in the mRNA expression of the stimulator of interferon genes was determined to be one of the molecular mechanisms underlying this suppression. Thus, the hepatic metabolic conversion of siphonaxanthin generates an **α**,**β**-unsaturated carbonyl group, which boosts its IRF-inhibitory effect by activating Nrf2.

Many phytochemicals are low-molecular-weight secondary metabolites and, therefore, potent nutraceuticals. Many researchers have screened phytochemicals for their biological activities using *in vitro* or cultured cell models. However, several phytochemicals have been reported to be metabolized *in vivo*, which may alter their biological activity. Nakai *et al.* (1) reported that although sesamin has no antioxidative effect, its demethylated metabolites produced in the mammalian liver exhibit strong antioxidative activity. In addition, Kure *et al.* (2) showed that conjugation with glucuronide decreased the anti-inflammatory activity of luteolin. Although its biological significance has not been fully elucidated, orally ingested phylloquinone is partially converted into menadione and menaquinone-4 in the intestinal epithelium (3). These reports indicate that research on the metabolic conversion of phytochemicals is vital for understanding and effectively utilizing their biological activities.

Carotenoids are lipophilic phytochemicals that are yellow to red in color. Thus far, more than 1000 carotenoids have been identified (4), and numerous studies have demonstrated their nutritional and health benefits. Carotenoid metabolism in mammals has been extensively investigated. The metabolic conversion of some carotenoids has been reported to alter their biological activity. β-Carotene is metabolically converted to vitamin A. In addition, fucoxanthinol, a deacetylated product of fucoxanthin (5), is metabolized to amarouciaxanthin A in mice, and its antiproliferative effect on human prostate cancer cells is decreased by this metabolic conversion (6). Nagao *et al.* (7) showed that the 3-hydroxy β-end group of carotenoids is enzymatically converted to a 3-oxo ε-end group in mice and humans (*e.g.*, β-cryptoxanthin, 3-hydroxy-β,β-carotene, is enzymatically converted to β,ε-caroten-3′one), which confers anti-inflammatory activities to 3-hydroxy β-end carotenoids.

Siphonaxanthin (3,19,3′-trihydroxy-7,8-dihydro-β,ε-caroten-8-one) is a green algal carotenoid with potent biological activities (8). It exhibits a more potent anti-inflammatory activity than other carotenoids (9, 10). We previously demonstrated that orally administered siphonaxanthin is absorbed from the small intestine (11) and distributed to various organs in mice (12, 13). Moreover, we also detected some unknown peaks in an HPLC-photodiode array (PDA) chromatogram of organ extracts derived from siphonaxanthin-fed mice (13, 14). The PDA spectra indicated that these unknown peaks could be attributed to siphonaxanthin-related compounds; however, their chemical structures and biological activities were unclear. In this study, we prepared three different siphonaxanthin metabolites by incubating mouse or rat livers with siphonaxanthin and characterized their chemical and biological properties. In addition, we elucidated the molecular mechanisms underlying the anti-inflammatory effects of siphonaxanthin and its metabolites by focusing on the differences in their chemical structures.

## Results

### NAD^+^-dependent dehydrogenation and isomerization of siphonaxanthin by rodent liver homogenates

**Figure 1:**
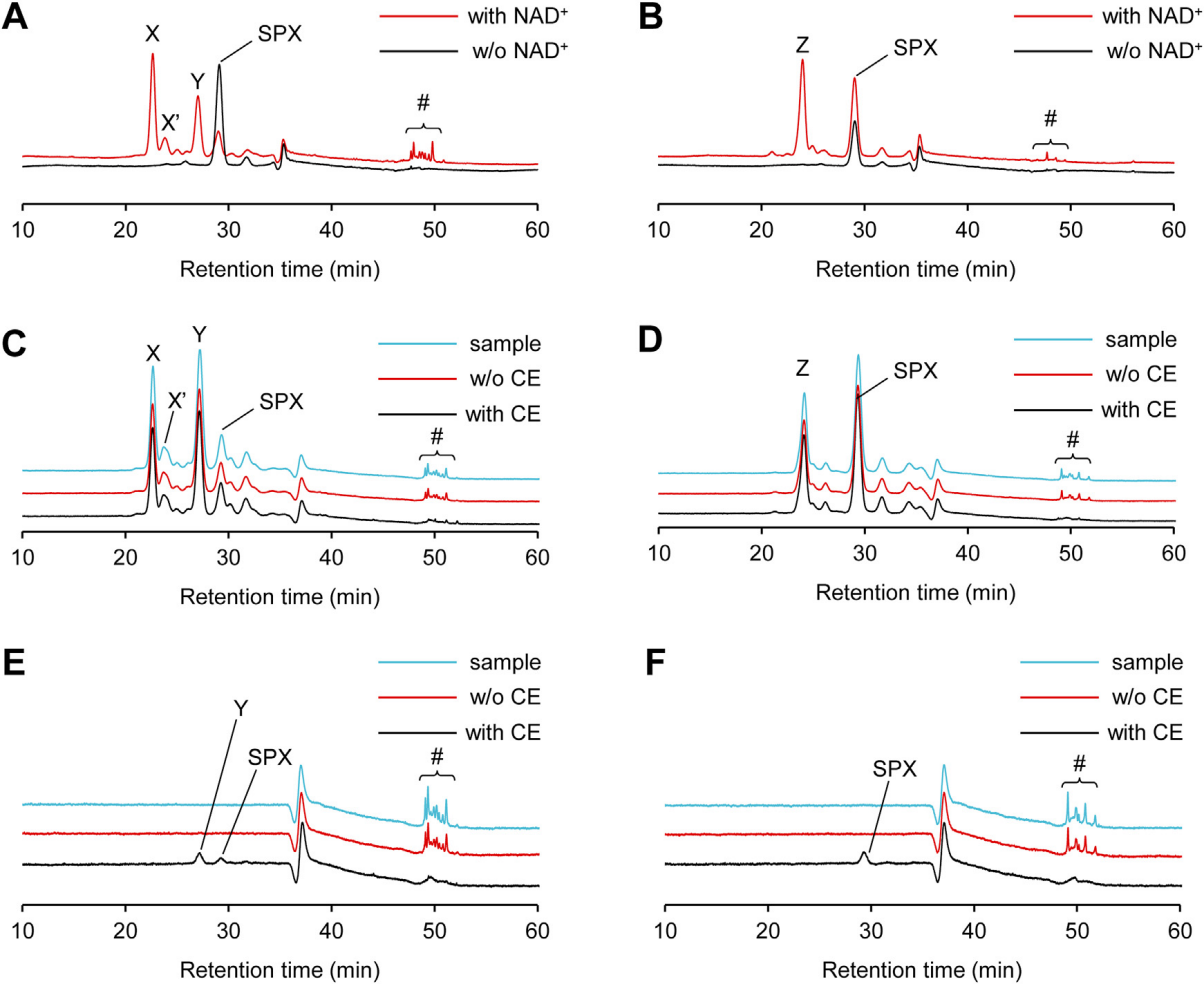
**HPLC-PDA chromatograms of carotenoids in incubation mixtures of siphonaxanthin (SPX) and rodent liver postmitochondrial supernatants (PMSs).** SPX was mixed with mouse liver PMS (*A*) or rat liver PMS (*B*). *C* and *D*, the lipophilic products of incubation of SPX and NAD^+^ with the mouse (*C*) or rat (*D*) liver PMS were further incubated with cholesterol esterase. Sample refers to the extracted lipids before cholesterol esterase treatment. *E* and *F*, peak # of C (*E*) and *D* (*F*) was fractionated and treated with cholesterol esterase. Sample refers to fractionated peak # before cholesterol esterase treatment. The chromatograms were monitored at 450 nm. CE, cholesterol esterase; PDA, photodiode array.

Mouse liver postmitochondrial supernatant (PMS) enzymatically converts the 3-hydroxy β-end group of carotenoids into a 3-keto ε-end group in an NAD^+^-dependent manner (4). Because siphonaxanthin has one 3-hydroxy β-end group, we incubated siphonaxanthin with mouse liver PMS in the presence or the absence of NAD^+^ for 24 h. HPLC-PDA chromatograms (λ = 450 nm) revealed the presence of four additional peaks (X, X′, Y, and #) only in the presence of NAD^+^ (Fig. 1*A*). Peak X showed an absorption maximum at 457 nm and had a spectrum identical to that of siphonaxanthin, indicating that it had the same chromophore system as siphonaxanthin. PDA and mass spectrometry (MS) spectra revealed that peak X′ is a *cis* isomer of peak X. Peak Y showed the same absorption maximum and PDA spectrum as peak X, but its MS spectrum was different from that of peak X and siphonaxanthin. Peak # disappeared after incubation with cholesterol esterase, indicating that it contained fatty acid esters of carotenoids (Fig. 1*C*). Incubation of fractionated peak # with cholesterol esterase revealed that peak # were fatty acid esters of peak Y and siphonaxanthin (Fig. 1*E*). We then conducted the same *in vitro* reaction using PMS from rat liver. A new peak, Z, was detected in addition to the siphonaxanthin peak in the presence but not in the absence of NAD^+^ (Fig. 1*B*). The PDA and MS spectra of peak Z were similar to those of peak Y. Peak # corresponded to fatty acid esters of siphonaxanthin but not of peak Z (Fig. 1, *D* and *F*), similar to the results obtained using mouse liver.

**Figure 2:**
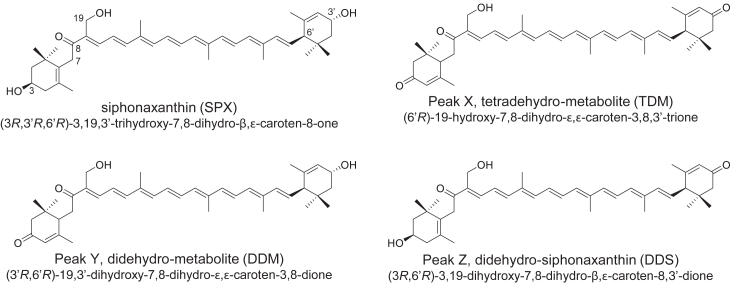
**Chemical structures of siphonaxanthin and its metabolites (peaks X–Z).** The three letters in parentheses are the corresponding abbreviations used in subsequent figures.

The MS spectra of the carotenoids from peak X showed an ion at *m/z* 597.3970 [M + H]^+^, indicating a molecular formula of C_40_H_52_O_4_, corresponding to a molecular weight four mass units less than that of siphonaxanthin. These mass spectral data suggested that the two hydroxy groups of siphonaxanthin were converted into two carbonyl (keto) groups. The ^1^H-NMR spectral data assigned using COSY and NOESY experiments for this compound are shown in Table S1. The two hydroxylated methin signals δ 4.01 (H-3′) and δ 4.26 (H-3) observed with siphonaxanthin were absent in this compound, indicating that the two hydroxyl groups were converted to carbonyl groups. The ^1^H-NMR signals of H-2′ to H-8′ and H-16′ to H-18′ also suggested the presence of a 3-oxo (keto)-ε-end group (15). The signals of the remaining end groups (H-2 to H-8 and H-16 to H-18) were assigned using COSY and NOESY experiments. The two AB-type doublet signals at δ 2.13 (*J* = 16 Hz) and δ 2.35 (*J* = 16 Hz) were assigned to the two methylene signals of H-2, and the broad singlet signal at δ 5.88 was assigned to the methin signal at H-4. A double doublet signal at δ 2.98 was assigned to the methin signal at H-6, and signals for the methyl groups at H-16, H-17, and H-18 were assigned based on NOE and long-range coupling between H-4 and H-18. From the ^1^H-NMR data, the structures of the end groups (C-1–C-6 and C-16–C-18) were determined to be a 3-oxo (keto)-ε-end group attached to a 7-hydro-8-keto-polyene chain. Therefore, the structure of carotenoid peak X was determined to be 19-hydroxy-7,8-dihydro-ε,ε-carotene-3,8,3′-trione (tetradehydrometabolite [TDM]; Fig. 2).

**Figure 3:**
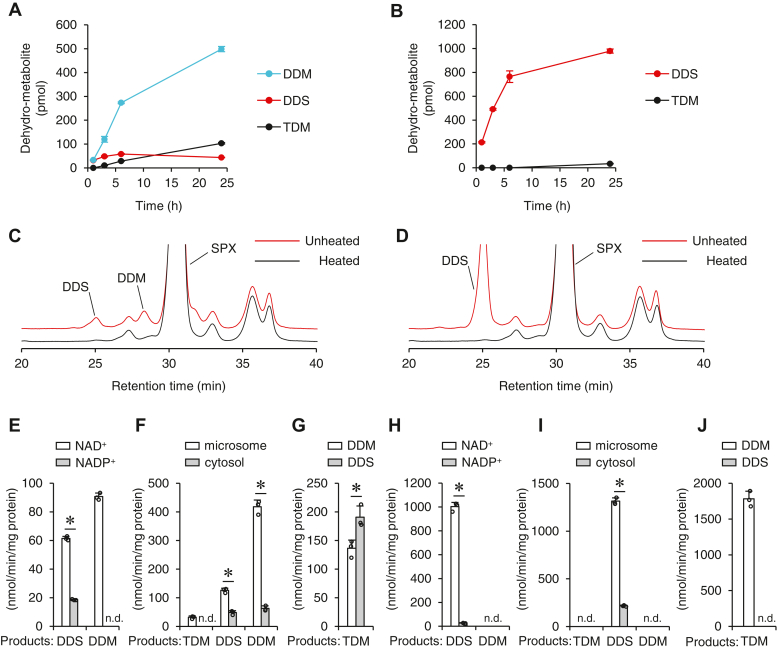
**Characterization of metabolic conversion of siphonaxanthin (SPX) by rodent liver S9 fraction.** Mouse (*A*) or rat (*B*) liver S9 fraction (0.2 mg protein) was incubated with SPX and NAD^+^ for the indicated time. *C* and *D*, mouse (*C*) or rat (*D*) liver S9 fraction was heated for 5 min at 95 °C and then incubated with SPX and NAD^+^ for 3 h. *E* and *H*, mouse (*E*) or rat (*H*) liver S9 fraction was incubated with SPX in the presence of either NAD^+^ or NADP^+^ for 3 h. *F* and *I*, mouse (*F*) or rat (*I*) liver microsome or cytosol was incubated with SPX and NAD^+^ for 3 h. *G* and *J*, mouse (*G*) or rat (*H*) liver S9 fraction was incubated with the indicated dehydrometabolite and NAD^+^ for 3 h. The chromatograms were monitored at 450 nm. Values represent mean ± SD of three technical replicates. The asterisk ∗ indicates significant differences (*p* < 0.05, Student’s unpaired *t* test). DDM, didehydrometabolite; DDS, didehydrosiphonaxanthin; ND, not detected; TDM, tetradehydrometabolite.

Similarly, the structures for peaks Y and Z were determined to be 19,3′-dihydroxy-7,8-dihydro-ε,ε-carotene-3,8-dione (didehydrometabolite [DDM]; Figs. 2) and 3,19-dihydroxy-7,8-dihydro-β,ε-carotene-8,3′-dione (didehydrosiphonaxanthin [DDS]; Fig. 2), respectively, using ^1^H-NMR and MS spectral data. The MS spectra of peaks Y and Z showed ions at *m/z* 581.4002 [M + H – H_2_O]^+^ and *m/z* 581.3992 [M + H – H_2_O]^+^, respectively, indicating a molecular formula of C_40_H_54_O_4_, which is two mass units less than that of siphonaxanthin. These mass spectral data suggest that one hydroxy group of siphonaxanthin was converted to a carbonyl (keto) group in these molecules. The ^1^H-NMR spectrum of peak Y is shown in Table S1. The signals of H-2 to H-6 and H-16, 17, and 18 were identical to those of 9-hydroxy-7,8-dihydro-ε,ε-carotene-3,8,3′-trione. The signals of another end group (H-2′ to H-6′ and H-16′ to 18′) were also identical to those of siphonaxanthin. Therefore, these spectral data suggest that the hydroxyl group at C-3 in siphonaxanthin was oxidized to a carbonyl group. Thus, the structure of peak Y was assigned to DDM. In contrast, carotenoids from peak Z showed the presence of the siphonaxanthin structural moiety of H-2 to H-6 and H-16 to H-18 and a 3-oxo (keto)-ε-end group (H-2′ to H-6′ and H-16′ to H-18′). Therefore, the structure of the Z peak was assigned to DDS.

CD analysis of the dehydrometabolites of siphonaxanthin was performed. TDM had a CD spectrum of 230 (Δε 0), 260 (+16), 299 (+1), 318 (+9.9), and 350 nm (0); DDS had a CD spectrum of 230 (Δε 0), 265 (+20), 294 (+3), 318 (+7), and 350 nm (0); and DDM exhibited a CD spectrum of 240 (Δε 0), 263 (+4), 271 (+4), 294 (0), 313 (+2), and 350 nm (0). These spectra indicated that TDM, DDS, and DDM had a positive Cotton effect (maximum) at 260 to 280 nm. Carotenoids with 6R or 6′R, or both, chirality with an ε-end group show a positive Cotton effect (maximum) in their CD spectrum at 260 to 280 nm (16). Hence, we concluded that these compounds have a 6′R chirality. The complete chemical structures of the siphonaxanthin dehydrometabolites are presented in Figure 2.

### Characterization of siphonaxanthin conversion to the dehydrometabolites

Mouse and rat liver S9 fractions were used to obtain further information regarding the bioconversion of siphonaxanthin. In mice, in addition to DDM and TDM, DDS was detected after a 24 h incubation of siphonaxanthin with the liver S9 fraction (Fig. 3*A*). After an hour of incubation, the amounts of DDM and DDS were identical; however, unlike DDM, DDS increased marginally with an increase in the incubation period. TDM appeared after 3 h of incubation and increased thereafter. In contrast, siphonaxanthin incubation with the rat liver S9 fraction produced large amounts of DDS, but no DDM (Fig. 3*B*), and a small amount of TDM after 24 h. In both cases, DDM and DDS increased linearly for up to at least 6 h of incubation; hence, the incubation time was set to 3 h for subsequent experiments. When siphonaxanthin was incubated with boiled S9 fractions, neither DDM nor DDS was produced (Fig. 3, *C* and *D*). NAD^+^ was the preferred cofactor over NADP^+^ for siphonaxanthin conversion to DDM and DDS in mouse and rat liver S9 fractions (Fig. 3, *E* and *H*). The dehydrogenation activity was higher in the microsomes than in the cytosol for both mouse and rat liver fractions (Fig. 3, *F* and *I*). Both DDM and DDS were converted to TDM by the mouse S9 fraction; DDS was the most productive substrate (Fig. 3*G*). In contrast, the rat liver S9 fraction converted DDM to TDM but not DDS (Fig. 3*J*).

### Human liver extracts and cell lines metabolized siphonaxanthin to DDM, DDS, or TDM

**Figure 4:**
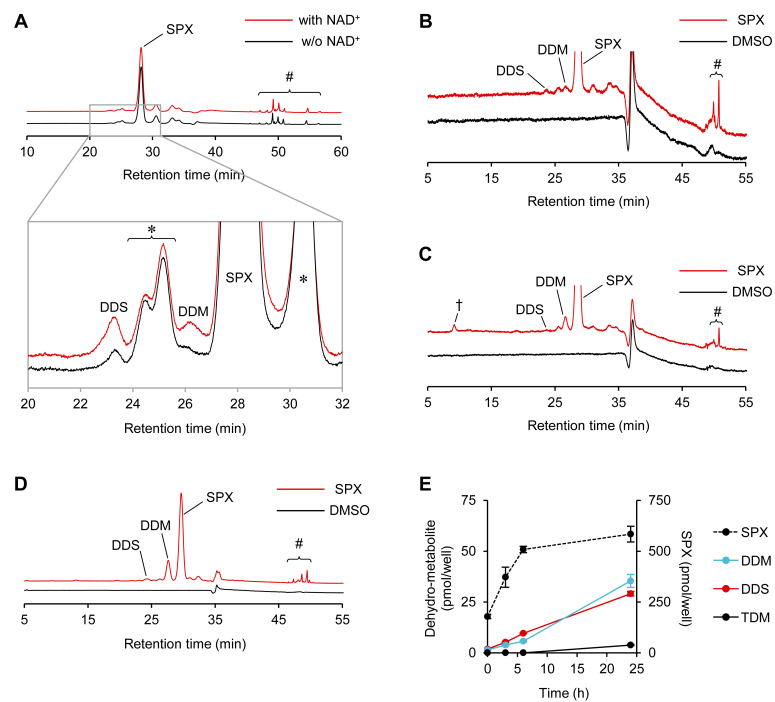
**Metabolic conversion of siphonaxanthin (SPX) by human liver S9 fraction and cell lines.** Human liver S9 fraction (*A*), HepaRG cells 24 h after seeding (*B*), HepaRG cells 7 days after seeding (*C*), HepG2 cells (*D*), and differentiated Caco-2 cells (*E*) were incubated with 1.0 μM SPX for 24 h (*A*–*D*) or the indicated periods (*E*). *A*–*D*, the chromatograms were monitored at 450 nm. The *lower panel* of *A* is a magnified chromatogram of the upper one. The asterisk ∗, pound #, and dagger † indicates *cis*-isomer, fatty acid ester, and an unidentified metabolite of SPX, respectively. *C*, the carotenoids were quantified from standard curves. Values represent mean ± SD of three biological replicates. DDM, didehydrometabolite; DDS, didehydrosiphonaxanthin; TDM, tetradehydrometabolite.

Because siphonaxanthin dehydrogenation and isomerization reactions differ between mice and rats, we incubated siphonaxanthin with a human liver S9 fraction in the presence or the absence of NAD^+^ to evaluate its metabolism in humans. Small amounts of DDM and DDS were generated from the human S9 fraction in an NAD^+^-dependent manner (Fig. 4*A*). HepaRG cells are a widely used human hepatocyte model for xenobiotic metabolism (17). According to the source instructions, the expression of the xenobiotic-metabolizing enzymes in cryopreserved differentiated HepaRG cells is dependent on the postseeding incubation time; cultures used within 24 h and after 7 days of seeding were most suitable for the experiments. Figure 4, *B* and *C* shows representative HPLC chromatograms of lipid extracts of HepaRG cells incubated with 2 nmol siphonaxanthin for either 24 h or 7 days after seeding, respectively. HepaRG cells 24 h after seeding generated 1.11 ± 0.239 DDS and 1.70 ± 0.263 pmol DDM, respectively, whereas HepaRG cells 7 days after seeding generated 1.03 ± 0.197 DDS and 6.48 ± 0.648 pmol DDM, respectively. A new peak, †, was detected. This showed absorption maxima and PDA spectra similar to those of siphonaxanthin (Fig. 4*C*). We also evaluated siphonaxanthin metabolism in HepG2 cells, a human hepatoma cell line. During a 24 h incubation period with 2 nmol of siphonaxanthin, HepG2 cells generated DDM (121 ± 5.62 pmol) and small amounts of DDS (13.8 ± 1.71 pmol, Fig. 4*D*). We analyzed the cellular accumulation of dehydrometabolites in differentiated Caco-2 cells, a model for the human intestinal epithelium since the intestinal epithelium, as well as the liver, can metabolize phytochemicals (3). Differentiated Caco-2 cells produced dehydrometabolites in an incubation time–dependent manner and generated 35.4 ± 3.19, 29.1 ± 1.15, and 3.82 ± 0.258 pmol of DDM, DDS, and TDM, respectively, when subjected to 1.6 nmol of siphonaxanthin-containing micelles for 24 h (Fig. 4*E*).

### Anti-inflammatory activity of siphonaxanthin and its dehydrometabolites

**Figure 5:**
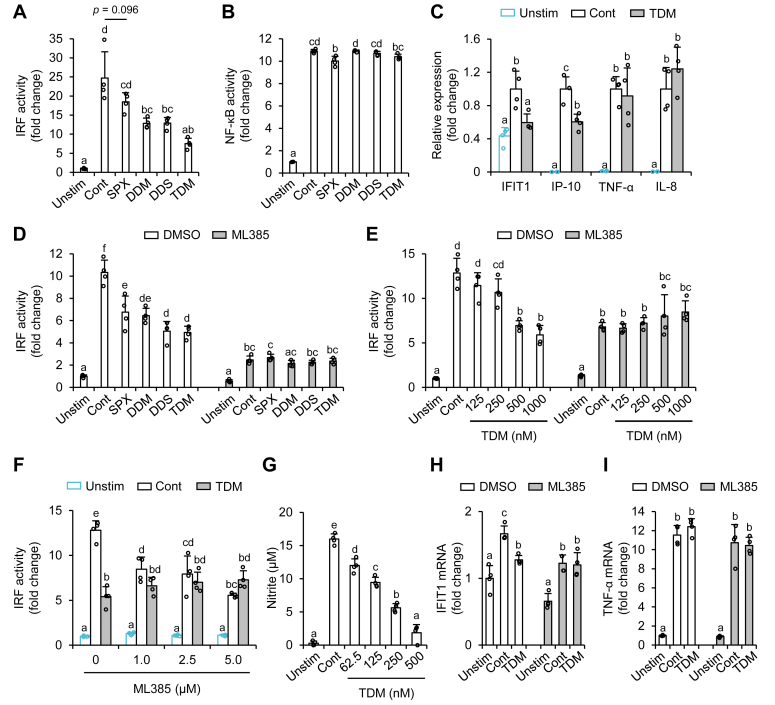
**Anti-inflammatory activities of siphonaxanthin (SPX) and its metabolites.***A*–*C*, THP1-Dual cells were treated with SPX or its metabolites (1 μM) for 4 h, followed by P3C stimulation. *D*–*F*, the cells were treated with the indicated carotenoids (1.0 μM for *D* and *F*; indicated concentrations for *E*) in the presence or the absence of ML385 (5.0 μM for *D* and *E*, indicated concentrations for *F*) for 4 h, followed by P3C stimulation. After 24 h of stimulation, luciferase (*A*, and *D*–*F*) and SEAP (*B*) activities in the supernatant were measured. In *C*, the cells were stimulated with P3C for 2 h, and RT–PCR was used to evaluate the mRNA expression of each gene. *G*, RAW264 cells were treated with the indicated concentration of TDM for 4 h, followed by P3C stimulation. After 24 h of stimulation, nitrite levels in the supernatant were measured. *H* and *I*, RAW264 cells were treated with 125 nM TDM in the presence or the absence of ML385 (5.0 μM) for 4 h. The treated cells were then stimulated with P3C for 6 h, and RT–PCR was used to evaluate the mRNA expression of each gene. Dimethyl sulfoxide was used as a vehicle (final concentration, 0.1%). Values represent mean ± SD of four biological replicates. The different characters represent significant differences among the treatments (*p* < 0.05, Tukey–Kramer’s test). Cont, control (P3C stimulation); DDM, didehydrometabolite; DDS, didehydrosiphonaxanthin; IFIT1, interferon-induced proteins with tetratricopeptide repeats 1; IL-8, interleukin-8; IP-10, 10 kDa interferon gamma-induced protein; IRF, interferon regulatory factor; P3C, Pam₃CSK₄; TDM, tetradehydrometabolite; TNF-α, tumor necrosis factor-α; SEAP, secreted embryonic alkaline phosphatase; Unstim, unstimulated

We previously reported that the keto group at the C-8 position is a key chemical structure involved in the anti-inflammatory activity of carotenoids (10, 18). In particular, C-8-keto-carotenoids suppress activation of interferon regulatory factor (IRF) induced by triacylated lipopeptide (Pam₃CSK₄ [P3C]) stimulation (10). Since all three dehydrometabolites of siphonaxanthin have a C-8-keto structure, we first evaluated their effects on P3C-induced IRF activation using a reporter gene assay. THP1-Dual cells are THP-1 human monocytes expressing secreted embryonic alkaline phosphatase and luciferase under the control of NF-κB and IRF, respectively. The dehydrometabolites of siphonaxanthin significantly suppressed, and siphonaxanthin tended to suppress IRF activation in P3C-exposed THP1-Dual cells (Fig. 5*A*). Similar trends in NF-κB activation or 3-(4,5-di-methylthiazol-2-yl)-2,5-di-phenyltetrazolium bromide reductase activity, a surrogate marker of cell viability, were not observed (Figs. 5*B* and S2*A*). In addition, there was no significant difference in the cellular accumulation of siphonaxanthin or its dehydrometabolites (Fig. S2*B*). Therefore, the differences in the inhibitory effects of siphonaxanthin and its dehydrometabolites may not be due to differences in their cytotoxicity or cellular accumulation. Similar to our previous study (10), we evaluated the effects of the metabolites on other stimulant-induced IRF, NF-κB activation, and 3-(4,5-di-methylthiazol-2-yl)-2,5-di-phenyltetrazolium bromide reductase activity (Fig. S2, *B*–*W*). The suppression of inflammatory responses by dehydrometabolites was not stronger than that observed with siphonaxanthin, except that the inhibitory effect of TDM on lipopolysaccharide (LPS)-induced IRF activation tended to be stronger than that of siphonaxanthin (Fig. S2*F*). Tumor necrosis factor-α (TNF-α)– and interleukin-1β (IL-1β)–induced NF-κB activation showed similar trends, but the inhibitory effects were small (Fig. S2, *J* and *M*).

Because TDM potently suppressed P3C-induced IRF activation, we attempted to confirm the reporter gene data using quantitative RT–PCR. TDM pretreatment significantly suppressed P3C-induced mRNA expression of interferon (IFN)-induced proteins with tetratricopeptide repeats 1 (IFIT1) and 10 kDa IFN gamma-induced protein (IP-10), which are downstream of IRF activation (Fig. 5*C*). In contrast, TDM pretreatment did not suppress P3C-induced TNF-α and IL-8 mRNA expression, both of which are regulated by NF-κB.

Since dehydrometabolites, but not siphonaxanthin, have an α,β-unsaturated carbonyl group (Fig. 2), which is one of the key chemical structures for the activation of nuclear factor erythroid 2–related factor 2 (Nrf2), we hypothesized that the activation of Nrf2 could be a molecular mechanism by which the dehydrometabolites of siphonaxanthin suppress P3C-induced IRF activation. The dehydrometabolites did not affect P3C-induced IRF activation in the presence of ML385, an Nrf2 inhibitor (Fig. 5*D*). TDM suppressed P3C-induced IRF activation in a dose-dependent manner but not in the presence of 5.0 μM ML385 (Fig. 5*E*). We did not observe similar trends in NF-κB activation (Fig. S3, *A* and *B*). AEM1, another Nrf2 inhibitor, showed similar effects to ML385 (Fig. S3, *C*–*F*). Since Nrf2 inhibitors (5.0 μM) potently suppressed P3C-induced IRF activation, we evaluated their effects at lower concentrations. Both ML385 and AEM1 significantly decreased IRF activity but not the NF-κB activity even at 1.0 μM (Figs. 5*F* and S3, *G*–*I*). At this concentration, Nrf2 inhibitors blunted the inhibitory effect of TDM (Figs. 5*F* and S3*H*).

Because the aforementioned biological data were collected using reporter gene–transfected cells, we also evaluated the effects of TDM on P3C-exposed RAW264 cells, a well-studied mouse macrophage model. TDM dose-dependently suppressed nitric oxide (NO) production, and 62.5 nM was sufficient to show the significant suppression (Fig. 5*G*). Both TDM and ML385 significantly decreased P3C-induced IFIT1 mRNA expression but did not significantly affect the mRNA expression of TNF-α (Fig. 5, *H* and *I*). The inhibitory effect of TDM on IFIT1 mRNA expression disappeared in the presence of ML385 (Fig. 5*H*).

### Nrf2–stimulator of interferon genes (STING) axis is crucial for the inhibitory effect of siphonaxanthin dehydrometabolites on P3C-induced IRF activation

**Figure 6:**
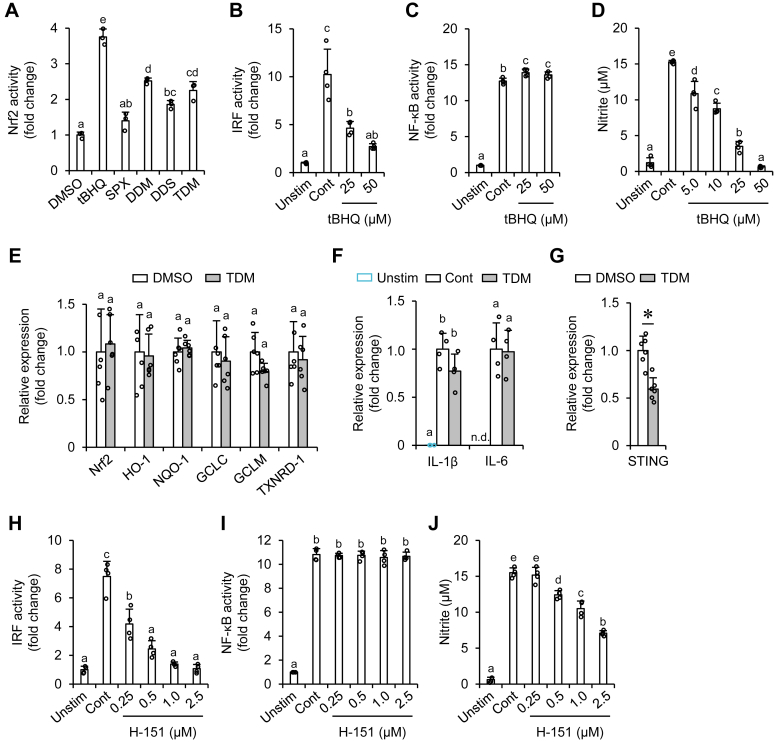
**The Nrf2–STING axis is essential for the inhibitory effect of dehydrometabolites of siphonaxanthin (SPX) against P3C-induced IRF activation.***A*, HepG2-ARE cells were treated with 100 μM of tBHQ or 5.0 μM of carotenoid. After 17 h of incubation, cellular luciferase activity was measured. *B*–*D* and *H*–*J*, THP1-Dual cells (*B* and *C*) and RAW264 cells (*D*) were treated with the indicated concentrations of tBHQ (*B*–*D*) or H-151 (*H*–*J*) for 4 h, followed by P3C stimulation. After 24 h of stimulation, luciferase (*B* and *H*), SEAP (*C* and *I*), and nitrite levels (*D* and *J*) in the supernatant were measured. *E*–*G*, THP1-Dual cells were treated with 1.0 μM of TDM for 4 h (*E* and *G*), followed by stimulation with P3C for 2 h (*F*). RT–PCR analysis was used to evaluate the mRNA expression of each gene. Dimethyl sulfoxide was used as a vehicle (the final concentration was 0.1%). Values represent mean ± SD of three (*A*), four (*B*–*D*, *F*, and *H*–*J*), and six (*E* and *G*) biological replicates. The asterisk ∗ indicates significant differences compared with the control. The different characters over the bar represent significant differences among the treatments (*p* < 0.05, Tukey–Kramer’s test for *A*–*D*, IL-1β in *F* and *H*–*J*, Student’s unpaired *t* test for *E*, IL-6 in *F* and *G*). Cont, control (P3C stimulation); DDM, didehydrometabolite; DDS, didehydrosiphonaxanthin; GCLC, glutamate–cysteine ligase catalytic subunit; GCLM, glutamate–cysteine ligase modifier subunit; HO-1, heme oxygenase-1; IL, interleukin; IRF, interferon regulatory factor; ND, not detected; NQO-1, NAD(P)H quinone dehydrogenase-1; Nrf2, nuclear factor erythroid 2–related factor 2; P3C, Pam₃CSK₄; STING, stimulator of interferon gene; tBHQ, *tert*-butylhydroquinone; TDM, tetradehydrometabolite; TXNRD-1, thioredoxin reductase-1; Unstim, unstimulated.

To clarify the effects of the metabolites on Nrf2 activation, we conducted an antioxidant response element (ARE) reporter gene assay. The dehydrometabolites, but not intact siphonaxanthin, significantly increased ARE-regulated luciferase expression (Fig. 6*A*). *Tert*-butylhydroquinone (tBHQ) was used as a positive control for Nrf2 activation (19) and significantly suppressed P3C-induced IRF but not NF-κB activation (Fig. 6, *B* and *C*). NO generation in P3C-exposed RAW264 macrophages was suppressed by tBHQ treatment (Fig. 6*D*), similar to that observed with TDM (Fig. 5*G*).

Figure 6*E* shows the mRNA expression of well-known Nrf2 target genes in THP1-Dual cells. Neither TDM treatment for 4 h (Fig. 6*E*) nor TDM pretreatment for 4 h, followed by P3C stimulation for 2 h (Fig. S4), increased the expression of Nrf2 target genes. Nrf2 has also been reported to block RNA polymerase II recruitment to the IL-1β and IL-6 loci and decreases their mRNA expression (20), yet TDM did not decrease P3C-induced mRNA expression of IL-1β or IL-6 (Fig. 6*F*). Olangnier *et al.* (21) reported that Nrf2 negatively regulates STING mRNA expression, and we found that TDM significantly decreased STING mRNA expression (Fig. 6*G*). Moreover, in addition to TDM and tBHQ, H-151, a selective inhibitor of STING (22), significantly suppressed P3C-induced IRF activation without affecting NF-κB activation (Fig. 6, *H* and *I*) in THP1-Dual cells and dose-dependently inhibited NO generation in P3C-exposed RAW264 macrophages (Fig. 6*J*).

## Discussion

To the best of our knowledge, this is the first report of the enzymatic conversion of a 3-hydroxy β-end group and a 3′-hydroxy ε-end group of siphonaxanthin to 3-keto and 3′-keto ε-end groups, respectively. In mouse liver, the initial velocity of the conversion from the 3-hydroxy β-end group to the 3-keto ε-end group was higher than that from the 3′-hydroxy ε-end group to the 3′-keto ε-end group. The conversion from DDM to TDM would be a rate-determining step from siphonaxanthin to TDM. In contrast, rat liver enzymatically converted the 3′-hydroxy ε-end group to a 3′-keto ε-end group, but the conversion of the 3-hydroxy β-end group to a 3-keto ε-end group barely occurred. These trends were also observed in conversions of DDS and DDM to TDM. In the human liver, either the 3-hydroxy β-end group or the 3′-hydroxy ε-end group was converted into the corresponding keto ε-end group. Thus, the enzymatic oxidation of 3- or 3′-hydroxy carotenoids in our diets, such as lutein, zeaxanthin, and β-cryptoxanthin, varies among species, and the possible metabolic conversions should be considered when extrapolating the results of animal studies to the health benefits of food ingredients in humans. In addition, it is essential to carefully select the appropriate animal species for an experimental model.

Dehydrogenation and isomerization of siphonaxanthin in the human liver appear to be much lower than those in mouse and rat livers. However, it may not be appropriate to compare them because the preparation methods and periods between the preparation and experiments were different. We freshly prepared liver samples for immediate use; however, the pooled human liver S9 fraction was purchased and frozen for an unknown period until use. According to our preliminary experiments, dehydrogenation and isomerization of siphonaxanthin decreased with storage time, even at −80 °C. Although many xenobiotic-metabolizing enzymes in HepG2 cells exhibit extremely low activity compared with those in human primary hepatocytes (23), HepG2 cells exhibited siphonaxanthin dehydrogenation and isomerization activity. In addition, differentiated Caco-2 cells, a widely used model for the human intestinal epithelium, generated DDMs and DDSs as well as TDMs from siphonaxanthin. Further investigations, including human trials, are required to clarify the extent to which siphonaxanthin is metabolized in the human body.

Although there may be lot-dependent differences, differentiated HepaRG cells express xenobiotic-metabolizing enzymes including cytochrome P450s at much higher levels than HepG2 cells (23). In this study, we found that HepG2 cells converted siphonaxanthin to DDM more effectively than HepaRG cells. Therefore, the enzymes reported to be more abundantly expressed in HepaRG cells than in HepG2 cells may not be involved in the conversion. Because conversion occurs at a relatively high pH and requires NAD^+^ as a cofactor, short-chain dehydrogenases may be responsible for the dehydrogenation of siphonaxanthin. Further studies are required to identify the enzymes involved in the process.

HepaRG cells produced an additional high polar metabolite (peak †). The absorption maximum indicated that it would not be a cleavage product of siphonaxanthin. HepaRG cells express both uridine diphosphate glucuronosyltransferase and sulfotransferase, and hence, peak † could be a glucuronide or a sulfate conjugate of siphonaxanthin or dehydrometabolites.

To obtain dehydrometabolites, we incubated siphonaxanthin with mouse or rat liver PMS and NAD^+^ (pH 9.5) for 24 h. Under these conditions, several unknown peaks (denoted as # in Fig. 1) were detected with a retention time of approximately 50 min. The results of cholesterol esterase treatments showed that they were the fatty acid esters of siphonaxanthin or DDM and not those of TDM or DDS. Mouse liver PMS generated TDM as much as DDM. The level of DDS generated by rat liver PMS was comparable to those of siphonaxanthin remained after the reaction. These results indicate that TDM and DDS were difficult to esterify using either mouse or rat liver PMS with NAD^+^ at pH 9.5. Therefore, a hydroxy group at C3′-position of the ε-end group would be easier to esterify with fatty acids than a hydroxy group at the C3-position of the β-end group under these experimental conditions.

We recently reported that the C-8-keto structure of carotenoids is an important factor for inhibiting P3C- and LPS-induced IRF activation (10). Consistent with this report, the dehydrometabolites of siphonaxanthin with their C-8-keto structures suppressed IRF activation. In some cases, the inhibitory effects of the dehydrometabolites were lower than those of siphonaxanthin; however, the differences were hardly visible. Hence, dehydrogenation and isomerization may not greatly weaken the anti-inflammatory effects of siphonaxanthin. Compared with siphonaxanthin, the dehydrometabolites showed a more potent inhibitory effect on the P3C-induced activation of IRF. Based on this structure–activity relationship, α,β-unsaturated carbonyl groups may be vital for the inhibitory effect. According to our results, the promotive effect on the transcription activation of Nrf2 was not proportional to the number of α,β-unsaturated carbonyl groups. Since the three dehydrometabolites significantly increased Nrf2 transcriptional activity, but siphonaxanthin did not, it is evident that the α,β-unsaturated carbonyl group is an essential chemical structure for this effect. However, the effect of DDM on Nrf2 activation in HepG2-ARE cells was significantly greater than that of DDS and marginally greater than that of TDM. HepG2 cells have siphonaxanthin dehydrogenase and acyltransferase; therefore, DDM and DDS might be metabolized to TDM or fatty acid esters or both in HepG2-ARE cells. In addition, HepG2 cells accumulated no TDM and less DDS than differentiated Caco-2 cells; hence, in HepG2-ARE cells, these dehydrometabolites could be unstable and perhaps further metabolized into unknown forms, possibly including degradation. The relationship between the number of α,β-unsaturated carbonyl groups and Nrf2 activation in metabolically active cells including hepatocytes should be investigated in detail in the future with a focus on further metabolism of the dehydrometabolites.

The relationship between Nrf2 and inflammation has been extensively investigated. For example, Nrf2 activation suppresses NF-κB activity by inducing heme oxygenase-1 (HO-1) expression (24). In this study, the dehydrometabolites of siphonaxanthin and tBHQ, an Nrf2 activator, did not suppress NF-κB activity in P3C-stimulated THP1-Dual cells, probably because the duration of treatment, the concentration used, or both were insufficient to induce the expression of antioxidant genes, including HO-1. The lack of suppression of IL-1β and IL-6 mRNA expression observed in this study may be due to the low concentration of TDM. However, our experimental conditions (treatment period and concentration) were sufficient for TDM to decrease the expression of STING mRNA, which has been reported to be destabilized by Nrf2 (21). STING activation leads to IRF activation (25), and we demonstrated that H-151, a potent and selective inhibitor of STING, significantly suppresses P3C-induced IRF activation. Therefore, we concluded that the dehydrometabolites of siphonaxanthin suppress IRF activation *via* the Nrf2–STING pathway.

The relationship between Nrf2 and IRF is complex. As mentioned previously, Nrf2 inhibits IRF activation by reducing STING expression. HO-1, whose expression is regulated by Nrf2, is required for IRF activation (26). Previous studies have shown that Nrf2 inhibitors significantly suppress P3C-induced IRF activation, which might be due to the reduction of HO-1 by Nrf2 inhibitors. The impact of simultaneous treatment with Nrf2 activators and inhibitors on the IRF activation is difficult to predict. The Nrf2 activator not only suppresses the IRF activation but also restores the suppressive effect of the Nrf2 inhibitor on the IRF activation and *vice versa*. In the case of treatment of ML385 at 5.0 μM and TDM at 1.0 μM, the suppression of IRF by TDM would be counteracted by ML385, and the inhibition of IRF by ML385 would be partially attenuated by TDM. This would be why the inhibitory rate of cotreatment of ML385 and TDM became smaller than that of treatment of ML385 alone. More detailed analysis would be required to understand the impact of simultaneous treatment with Nrf2 activators and inhibitors on the IRF activation.

We also evaluated the inhibitory effects of TDM using the P3C-exposed mouse macrophage cell line RAW264. Autocrine IFN-β, whose expression is regulated by IRF3 in toll-like receptor ligand–stimulated macrophages, induces inducible nitric oxide synthase expression and subsequent NO generation (27). Hence, the inhibitory effect of TDM on NO generation, measured as nitrite, resulted from the inhibition of IRF activation. TDM significantly inhibited IFIT1 mRNA expression but not in the presence of ML385. However, it did not affect the TNF-α mRNA expression. Therefore, TDM suppresses P3C-induced IRF activation *via* Nrf2 activation.

TDM significantly suppressed P3C-induced NO generation in RAW264 cells even at 62.5 nM. Considering the results of this study (identification of siphonaxanthin dehydrometabolites) with those of our previous animal feeding study (13), the concentrations of TDM, DDS, and DDM in the plasma were approximately 162, 3.5, and 147 nM, respectively. DDS may not but DDM might exhibit the anti-inflammatory effects *in vivo*. Whereas TDM inhibited P3C-induced NO generation at physiological concentrations.

In conclusion, we identified the chemical structures of the dehydrometabolites of siphonaxanthin that suppress toll-like receptor 1/2–mediated IRF activation *via* Nrf2 activation and the subsequent decrease in STING expression. This study shows that metabolic conversion can enhance biological activity and demonstrates the usefulness of *in vitro* enzymatic reactions and subsequent chemical analyses for investigating the biological activity of phytochemicals.

## Experimental procedures

### Reagents

Mouse and rat liver S9, microsomal and cytosolic fractions, and human liver S9 fraction (Xtreme 200 pooled human liver S9) were obtained from Sekisui Medical. LPS from *Escherichia coli* 0111:B4 was purchased from Santa Cruz Biotechnology. P3C and phorbol 12-myristate 13-acetate were obtained from Abcam. Polyinosinic–polycytidylic acid sodium salt, recombinant human IFN-γ, AEM1, and H-151 were purchased from Sigma–Aldrich. Recombinant human TNF-α and IL-1β were purchased from PeproTech. Advanced glycation end product (AGE [AGE–bovine serum albumin]) was purchased from BioVision. ML385 and tBHQ were purchased from Cayman Chemical Co and FUJIFILM Wako Pure Chemical Corporation, respectively. All other chemicals, media, and solvents used in the experiments were of commercially available grade.

### Preparation of siphonaxanthin

Siphonaxanthin was prepared from the green alga *Codium fragile* as previously described, with minor modifications (28). Briefly, the acetone extract of the algae was dissolved in hexane and subjected to open-column chromatography on silica gel. The column was eluted stepwise with 400 ml of 0, 10, 20, 30, and 40% acetone in hexane. The siphonaxanthin-rich fraction, eluted with 40% acetone in hexane, was further subjected to HPLC on a TSK gel ODS-80Ts column (4.6 mm × 250 mm, 5 μm; Tosoh) using a mobile phase of acetonitrile:methanol:water (75/15/10, v/v/v). Purified siphonaxanthin (purity > 95%) was stored at −80 °C until use.

### PMS preparation from mouse and rat liver

All the animal experiments were approved by the Animal Experimentation Committee of Kyoto University (approval no.: R2-96). Male 7-week-old ICR (Institute for Cancer Research) mice and male 7-week-old Sprague–Dawley rats were obtained from Japan SLC, Inc. All animals were housed individually and maintained at 23 ± 1.0 °C under a 12 h dark–light cycle with free access to drinking water and a standard chow diet (Oriental Yeast Co, Ltd). After an acclimatization period of 7 days, the animals were deprived of food for 16 h and sacrificed under isoflurane anesthesia. After perfusion with saline, the liver was removed and homogenized in a Potter–Elvehjem homogenizer with four volumes of 0.1 M glycine–KOH buffer (pH 9.5) containing 0.154 M KCl, 1.0 mM EDTA, 1.0 mM EGTA, and 0.5 mM DTT. PMS was obtained by centrifugation at 10,000*g* for 10 min.

### Siphonaxanthin metabolic conversion by rodent liver PMS, S9, microsome, cytosol, or human liver S9 fraction

The reaction mixtures contained 20 μM siphonaxanthin, 0.1 M glycine–KOH buffer, 2.4 mM NAD^+^, 1.0 mM EDTA, 1.0 mM EGTA, 0.5 mM DTT, and mouse or rat liver PMS (2.5 mg of protein), S9, microsomes, cytosol (0.2 mg of protein), or human liver S9 fraction (1.0 mg of protein) in a total volume of 0.1 ml. Tween-20 was used to solubilize siphonaxanthin, and the final concentration of Tween-20 was set at 0.2% of the total volume. The reactions were allowed to proceed at 37 °C in the dark for the indicated periods under shaking conditions. After the reaction period, 0.44 ml of Milli-Q water, 0.6 ml of methanol, and 1.2 ml of chloroform were added to the reaction mixtures to extract reaction products into the organic phase. After evaporating the solvent, the residues were dissolved in methanol and subjected to LC–MS analysis as described previously (13).

### Cholesterol esterase treatment

Carotenoid samples were incubated in 200 mM potassium phosphate buffer (pH 6.8) containing 10 mg/ml sodium taurocholate and 2.0 U/ml cholesterol esterase from *Pseudomonas* sp at 37 °C for 2 h. After the reaction period, the carotenoids were extracted and subjected to LC–MS analysis as described previously.

### Isolation of major siphonaxanthin metabolites

The lipid extracts from the reaction mixtures containing mouse liver PMS were applied to PLC silica gel 60 F254 plates (20 cm × 20 cm, 1.0 mm; Merck). Chloroform/ethyl acetate (70/30, v/v) was used as the developing solvent. Each yellow band was scraped off the plate and purified by silica gel HPLC using hexane/acetone (80/20, v/v) as the mobile phase. To isolate the metabolites produced by rat liver PMS, chloroform/ethyl acetate (80/20, v/v) and hexane/acetone (70/30, v/v) were used as the developing solvent and mobile phase, respectively. Purity was confirmed using LC–PDA–MS analysis (Fig. S1).

### Determination of the chemical structure of siphonaxanthin metabolites

^1^H-NMR (500 MHz) spectra were recorded using a UNITY INOVA-500 spectrometer (Varian) in CDCl_3_, with tetramethylsilane as the internal standard. CD spectra of the metabolites were measured in diethyl ether using a J-500C instrument (Jasco) at room temperature.

### Cell culture

Human hepatoma HepG2 cells, human adenocarcinoma Caco-2 cells, and mouse macrophage RAW264 cells were obtained from the RIKEN Gene Bank. THP1-Dual cells and ARE reporter-transfected HepG2 cells (HepG2-ARE) were purchased from InvivoGen and BPS Bioscience, respectively. HepaRG cells were purchased from KAC Co, Ltd. HepG2 cells were cultured in Dulbecco’s modified Eagle's medium. Caco-2 cells were maintained in Dulbecco’s modified Eagle's medium supplemented with 1.0% nonessential amino acids, and RAW264 cells were cultured in Roswell Park Memorial Institute 1640 medium. The medium contained 10% fetal bovine serum, 100 U/ml penicillin, and 100 μg/ml streptomycin. To induce differentiation, Caco-2 cells were cultured in 24-well plates at a density of 5.0 × 10^4^ cells/well for 21 days with medium change every 2 or 3 days. THP1-Dual, HepG2-ARE, and HepaRG cells were maintained according to the source instructions. The cell cultures were incubated at 37 °C in a humidified atmosphere containing 5.0% CO_2_.

### Carotenoid treatment

Dimethyl sulfoxide (final concentration 0.1%) was used as the vehicle to disperse siphonaxanthin and its metabolites into the culture medium. To treat differentiated Caco-2 cells with siphonaxanthin, we prepared mixed micelles as previously described (29).

### Dehydrogenation and isomerization of siphonaxanthin in HepG2, HepaRG, and differentiated Caco-2 cells

Ninety percent confluent HepG2 cells that adhered to 6-well plates were rinsed with serum-free medium and treated with a 1.0 μM siphonaxanthin-containing medium (2 ml). Differentiated HepaRG cells were seeded in type I collagen–coated 6-well plates. After 7 days of culture, the cells were treated with a 1.0 μM siphonaxanthin-containing medium (2.0 ml). For evaluating metabolic activity within 24 h of seeding, the cells were seeded in a 1.0 μM siphonaxanthin-containing medium (2.0 ml). Differentiated Caco-2 cells were treated with a serum-free medium containing 1.0 μM micellar siphonaxanthin (1.6 ml). After incubation for the indicated periods, total lipids were prepared from the cells as described previously (11). Carotenoid levels were quantified using LC–PDA–MS.

### Evaluation of anti-inflammatory activity

THP1-Dual cells plated at a density of 2.5 × 10^5^ cells/ml were treated with carotenoids for 4 h, followed by stimulation for 24 h. The concentrations of the stimulants were as follows: P3C, 100 ng/ml; polyinosinic–polycytidylic acid sodium salt, 100 μg/ml; LPS, 100 ng/ml; TNF-α, 100 ng/ml; IL-1β, 10 ng/ml; IFN-γ, 100 ng/ml; AGEs, 100 μg/ml; and phorbol 12-myristate 13-acetate, 100 ng/ml. To evaluate NF-κB activation, aliquots (20 μl) of the supernatant were mixed with QUANTI-Blue solution (100 μl). After 15 min of incubation at 37 °C, visible absorption at 640 nm was measured using a spectrophotometer. To analyze IRF activation levels, the luciferase activity of the supernatant (20 μl) was measured 4 s after the automated injection of QUANTI-Luc solution (50 μl) using a luminometer. RAW264 cells plated at a density of 5.0 × 10^5^ cells/ml were treated with carotenoids for 4 h, followed by stimulation with 100 ng/ml of P3C. After 24 h of incubation, nitrite levels in the supernatant were determined using the Griess reaction assay (9).

### RT–PCR analysis

THP1-Dual and RAW264 cells were treated with the indicated carotenoids for 4 h, followed by stimulation with 100 ng/ml P3C for 2 and 6 h, respectively. RT–PCR was performed as described previously (28). The primer sequences are listed in Table S2.

### Evaluation of Nrf2 activation activity

HepG2-ARE cells were seeded at a density of 4.0 × 10^4^ cells/well in a 96-well plate. The carotenoid-containing medium was added to each well, and the cells were incubated at 37 °C in a humidified atmosphere with 5.0% CO_2_. After incubating for 17 h, the luciferase activity was evaluated using Britelite Plus Reagent (PerkinElmer) according to the manufacturer’s instructions.

### Statistical analysis

All data are presented as mean ± SD. Statistical analyses were performed using a one-way ANOVA, followed by the Tukey–Kramer test, or Student’s unpaired *t* test. The results of ANOVA and *p* values of Student’s unpaired *t* test are presented in Tables S3 and S4, respectively.

## Data availability

All data are contained within the article and in the supporting information.

## Supporting information

This article contains supporting information.

## Conflict of interest

The authors declare that they have no conflicts of interest with the contents of this article.
