# Supplementary material for: Dehydrometabolites of siphonaxanthin, a carotenoid from green algae, suppress toll-like receptor 1/2-induced inflammatory response more strongly than siphonaxanthin
Source: J Biol Chem. 2025 Jan 31;301(3):108246. doi: 10.1016/j.jbc.2025.108246 (PMC11908587; doi:10.1016/j.jbc.2025.108246)
Supplement: Supplementary Figure [file mmc1.pptx]

## Slide 1
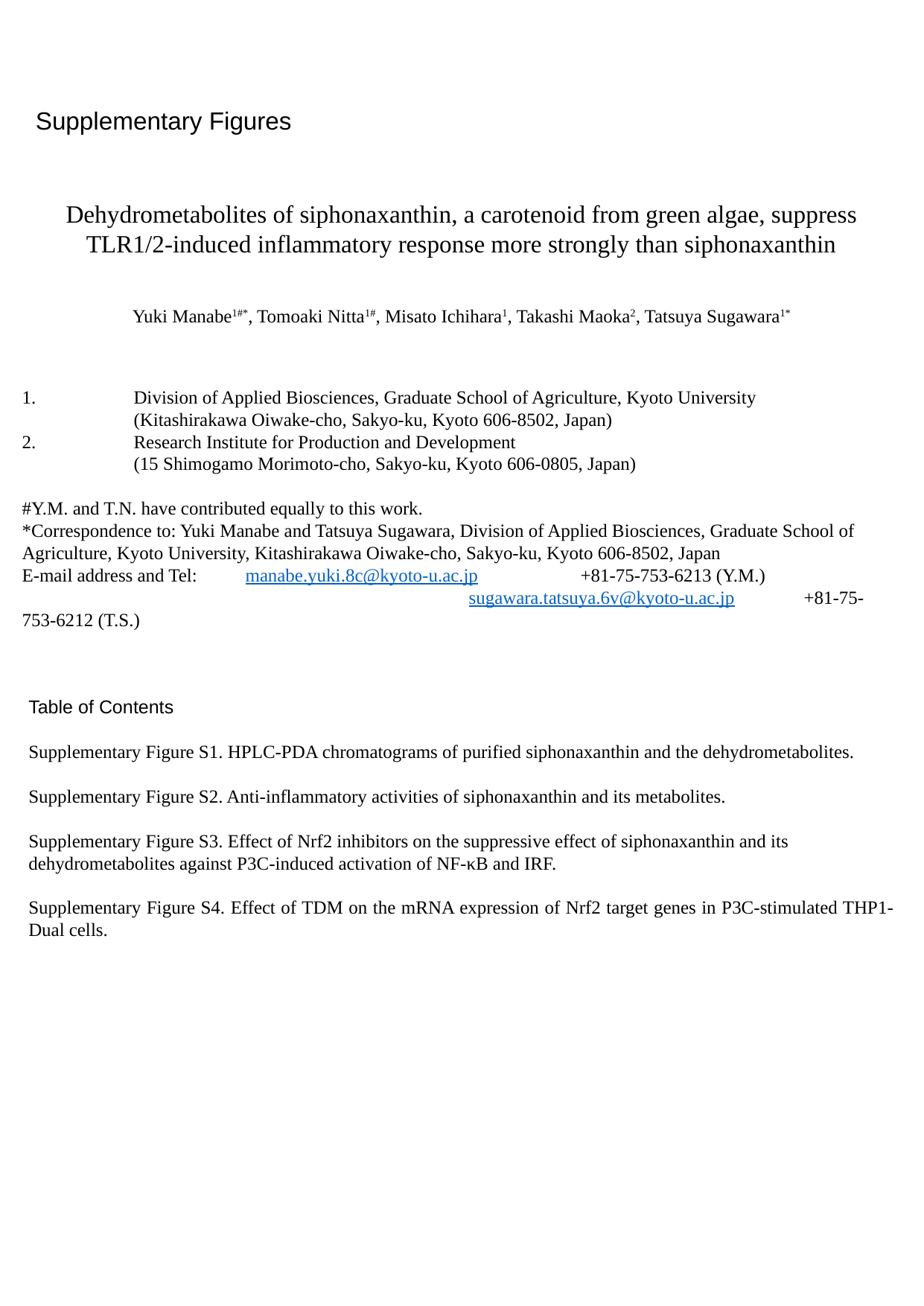

Supplementary Figures
Dehydrometabolites of siphonaxanthin, a carotenoid from green algae, suppress TLR1/2-induced inflammatory response more strongly than siphonaxanthin
Yuki Manabe1#*, Tomoaki Nitta1#, Misato Ichihara1, Takashi Maoka2, Tatsuya Sugawara1*
1.	Division of Applied Biosciences, Graduate School of Agriculture, Kyoto University
	(Kitashirakawa Oiwake-cho, Sakyo-ku, Kyoto 606-8502, Japan)
2.	Research Institute for Production and Development
	(15 Shimogamo Morimoto-cho, Sakyo-ku, Kyoto 606-0805, Japan)
#Y.M. and T.N. have contributed equally to this work.
*Correspondence to: Yuki Manabe and Tatsuya Sugawara, Division of Applied Biosciences, Graduate School of Agriculture, Kyoto University, Kitashirakawa Oiwake-cho, Sakyo-ku, Kyoto 606-8502, Japan
E-mail address and Tel:	manabe.yuki.8c@kyoto-u.ac.jp	+81-75-753-6213 (Y.M.)
				sugawara.tatsuya.6v@kyoto-u.ac.jp	+81-75-753-6212 (T.S.)
Table of Contents
Supplementary Figure S1. HPLC-PDA chromatograms of purified siphonaxanthin and the dehydrometabolites.
Supplementary Figure S2. Anti-inflammatory activities of siphonaxanthin and its metabolites.
Supplementary Figure S3. Effect of Nrf2 inhibitors on the suppressive effect of siphonaxanthin and its dehydrometabolites against P3C-induced activation of NF-κB and IRF.
Supplementary Figure S4. Effect of TDM on the mRNA expression of Nrf2 target genes in P3C-stimulated THP1-Dual cells.

## Slide 2
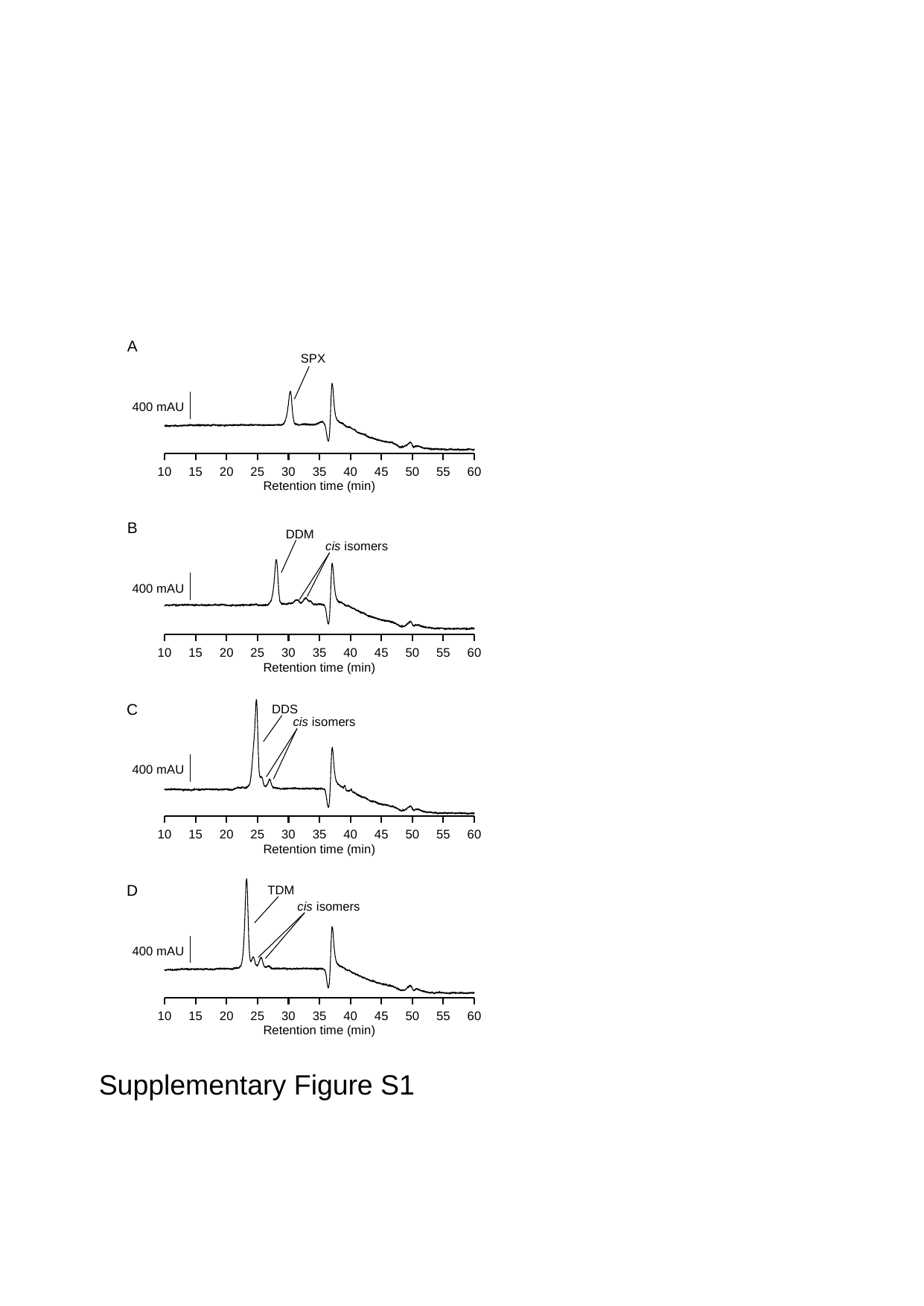

### Chart
| Category | |
|---|---|A
400 mAU
Retention time (min)
SPX
### Chart
| Category | |
|---|---|B
400 mAU
Retention time (min)
DDM
cis isomers
### Chart
| Category | |
|---|---|C
400 mAU
Retention time (min)
DDS
cis isomers
### Chart
| Category | |
|---|---|D
400 mAU
Retention time (min)
TDM
cis isomers
Supplementary Figure S1

## Slide 3
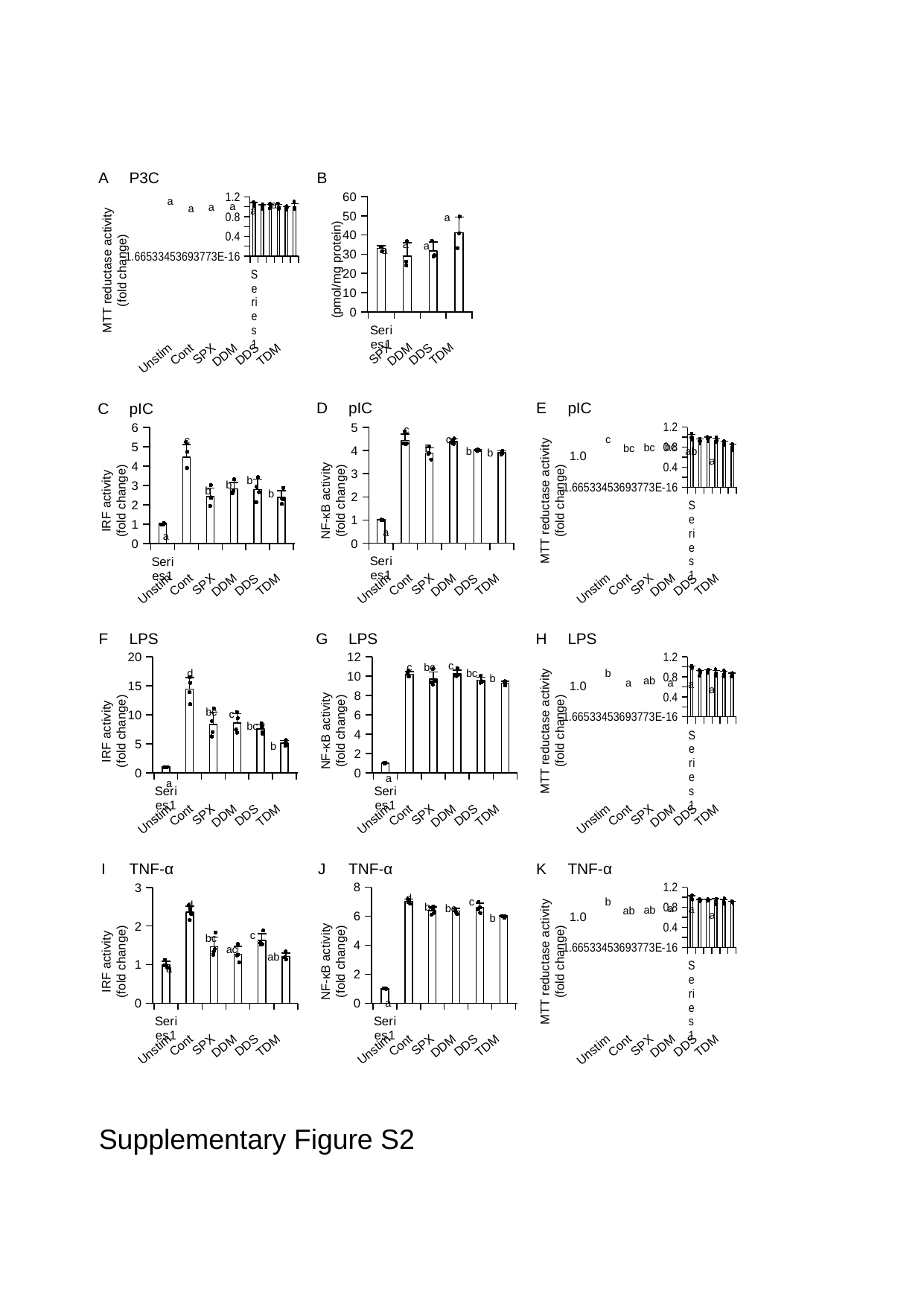

B
A
P3C
[unsupported chart]
[unsupported chart]
a
a
a
a
a
a
a
a
a
a
MTT reductase activity
(fold change)
(pmol/mg protein)
SPX
DDM
DDS
TDM
Unstim
Cont
SPX
DDM
DDS
TDM
D
pIC
E
pIC
C
pIC
[unsupported chart]
[unsupported chart]
[unsupported chart]
c
c
c
c
bc
bc
bc
b
b
ab
b
1.0
a
b
b
b
NF-κB activity
(fold change)
MTT reductase activity
(fold change)
IRF activity
(fold change)
b
a
a
Unstim
Cont
SPX
DDM
DDS
TDM
Unstim
Cont
SPX
DDM
DDS
TDM
Unstim
Cont
SPX
DDM
DDS
TDM
G
LPS
H
LPS
F
LPS
[unsupported chart]
[unsupported chart]
[unsupported chart]
c
bc
c
d
b
bc
b
ab
a
a
a
a
1.0
bc
c
NF-κB activity
(fold change)
MTT reductase activity
(fold change)
IRF activity
(fold change)
bc
b
a
a
Unstim
Cont
SPX
DDM
DDS
TDM
Unstim
Cont
SPX
DDM
DDS
TDM
Unstim
Cont
SPX
DDM
DDS
TDM
J
TNF-α
I
TNF-α
K
TNF-α
[unsupported chart]
[unsupported chart]
[unsupported chart]
d
b
c
d
bc
a
bc
a
ab
ab
a
b
1.0
c
bc
ac
NF-κB activity
(fold change)
IRF activity
(fold change)
MTT reductase activity
(fold change)
ab
a
a
Unstim
Cont
SPX
DDM
DDS
TDM
Unstim
Cont
SPX
DDM
DDS
TDM
Unstim
Cont
SPX
DDM
DDS
TDM
Supplementary Figure S2

## Slide 4
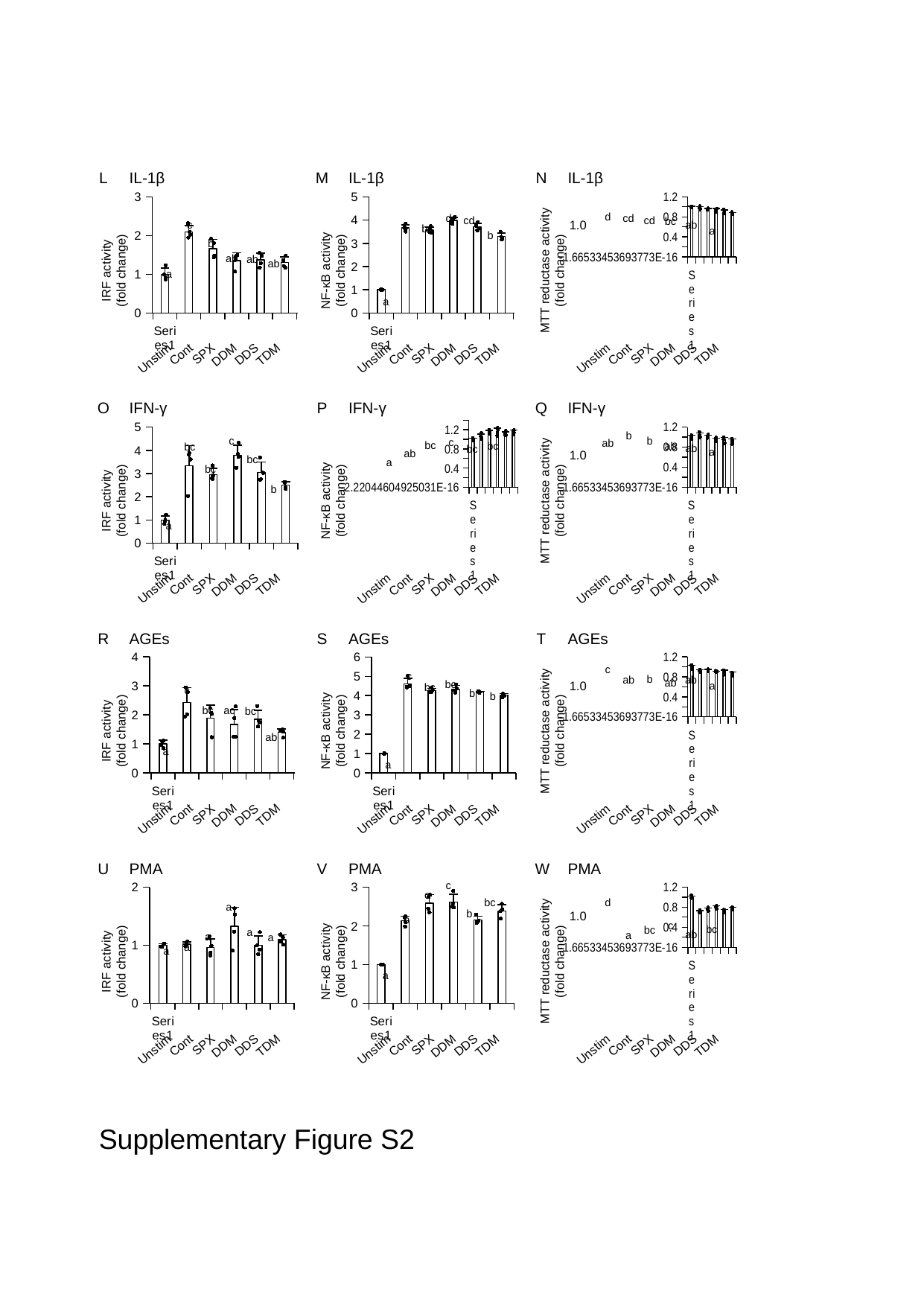

L
IL-1β
M
IL-1β
N
IL-1β
[unsupported chart]
[unsupported chart]
[unsupported chart]
d
cd
d
cd
cd
bc
ab
c
c
bc
1.0
a
b
b
ab
ab
IRF activity
(fold change)
NF-κB activity
(fold change)
MTT reductase activity
(fold change)
ab
a
a
Unstim
Cont
SPX
DDM
DDS
TDM
Unstim
Cont
SPX
DDM
DDS
TDM
Unstim
Cont
SPX
DDM
DDS
TDM
O
IFN-γ
P
IFN-γ
Q
IFN-γ
[unsupported chart]
[unsupported chart]
[unsupported chart]
b
c
b
c
ab
bc
ab
bc
bc
ab
bc
a
ab
bc
1.0
a
bc
b
IRF activity
(fold change)
NF-κB activity
(fold change)
MTT reductase activity
(fold change)
a
Unstim
Cont
SPX
DDM
DDS
TDM
Unstim
Cont
SPX
DDM
DDS
TDM
Unstim
Cont
SPX
DDM
DDS
TDM
R
AGEs
S
AGEs
T
AGEs
[unsupported chart]
[unsupported chart]
[unsupported chart]
c
c
b
ab
ab
ab
bc
a
bc
c
1.0
b
b
bc
ac
bc
IRF activity
(fold change)
NF-κB activity
(fold change)
MTT reductase activity
(fold change)
ab
a
a
Unstim
Cont
SPX
DDM
DDS
TDM
Unstim
Cont
SPX
DDM
DDS
TDM
Unstim
Cont
SPX
DDM
DDS
TDM
U
PMA
V
PMA
W
PMA
c
[unsupported chart]
[unsupported chart]
[unsupported chart]
c
bc
d
a
b
b
1.0
c
bc
bc
a
ab
a
a
a
a
a
IRF activity
(fold change)
NF-κB activity
(fold change)
MTT reductase activity
(fold change)
a
Unstim
Cont
SPX
DDM
DDS
TDM
Unstim
Cont
SPX
DDM
DDS
TDM
Unstim
Cont
SPX
DDM
DDS
TDM
Supplementary Figure S2

## Slide 5
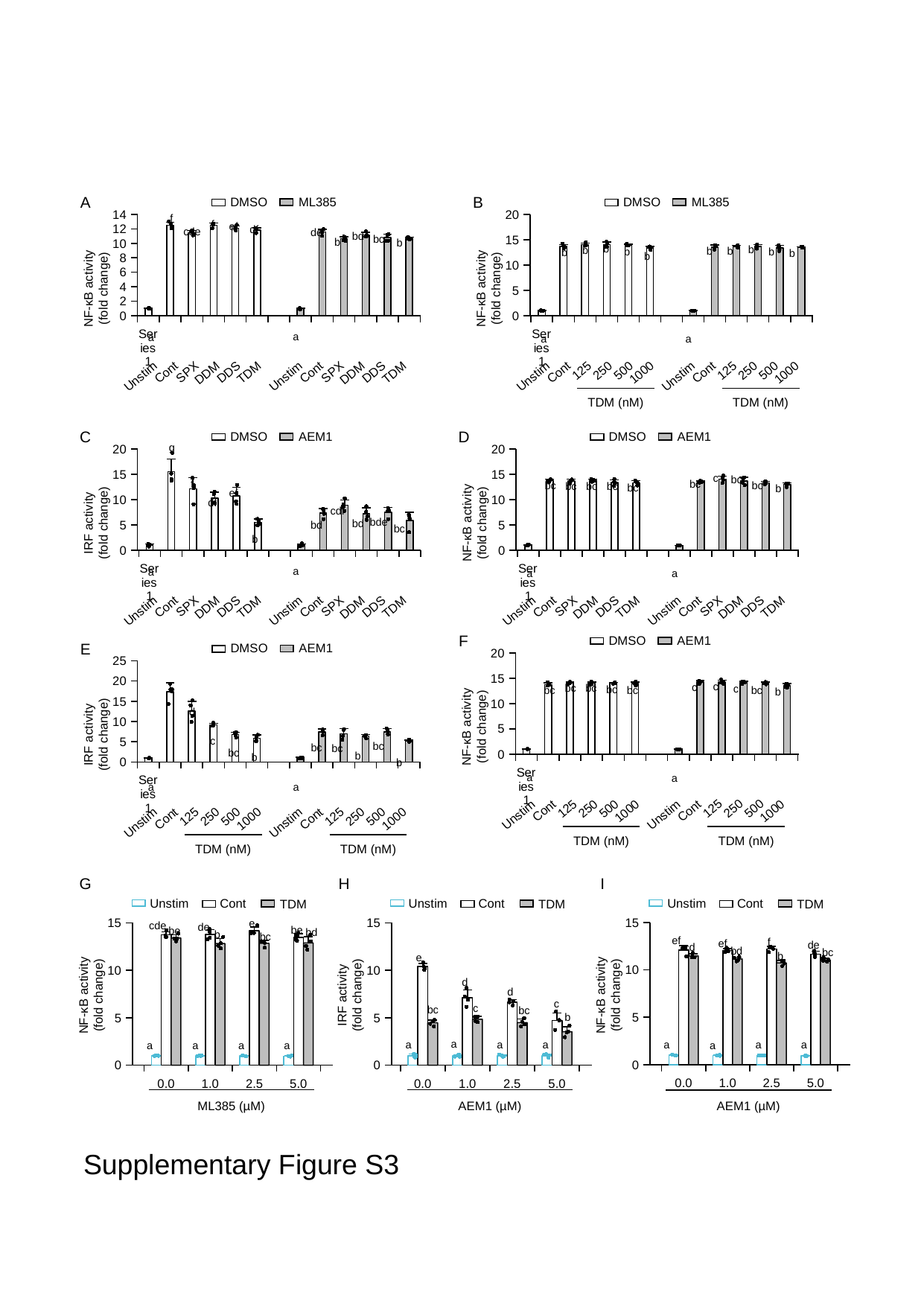

A
B
DMSO
ML385
DMSO
ML385
[unsupported chart]
[unsupported chart]
f
f
ef
df
cde
de
bd
bc
b
b
b
b
b
b
b
b
b
b
b
b
NF-κB activity
(fold change)
NF-κB activity
(fold change)
a
a
a
a
Unstim
Cont
SPX
DDM
DDS
TDM
Unstim
Cont
SPX
DDM
DDS
TDM
Unstim
Cont
125
250
500
1000
Unstim
Cont
125
250
500
1000
TDM (nM)
TDM (nM)
C
D
DMSO
AEM1
DMSO
AEM1
g
[unsupported chart]
[unsupported chart]
c
bc
f
bc
bc
bc
bc
bc
bc
bc
b
ef
df
cdf
IRF activity
(fold change)
NF-κB activity
(fold change)
bde
bd
bd
bc
b
a
a
a
a
Unstim
Cont
SPX
DDM
DDS
TDM
Unstim
Cont
SPX
DDM
DDS
TDM
Unstim
Cont
SPX
DDM
DDS
TDM
Unstim
Cont
SPX
DDM
DDS
TDM
F
DMSO
AEM1
E
DMSO
AEM1
[unsupported chart]
[unsupported chart]
c
c
bc
bc
c
bc
e
bc
bc
bc
b
d
NF-κB activity
(fold change)
IRF activity
(fold change)
c
bc
bc
bc
bc
b
b
b
a
a
a
a
Unstim
Cont
125
250
500
1000
Unstim
Cont
125
250
500
1000
Unstim
Cont
125
250
500
1000
Unstim
Cont
125
250
500
1000
TDM (nM)
TDM (nM)
TDM (nM)
TDM (nM)
G
H
I
Unstim
Cont
TDM
Unstim
Cont
TDM
Unstim
Cont
TDM
e
### Chart
| Category | | | | | | | | | | | | | | | |
|---|---|---|---|---|---|---|---|---|---|---|---|---|---|---|---|
| 0 | 1.0 | 12.09090909090909 | 11.467601547388782 | 0.9729206963249516 | 0.9535783365570598 | 0.9497098646034815 | 0.9400386847195357 | 11.406189555125724 | 12.317214700193423 | 11.864603481624757 | 11.446808510638297 | 11.390715667311412 | 11.394584139264989 | 10.972920696324952 | 10.83752417794971 |
| 1 | 0.9656673114119921 | 12.034816247582205 | 11.145067698259187 | 1.0270793036750483 | 0.9535783365570598 | 0.9651837524177949 | 0.9361702127659574 | 12.305609284332688 | 11.870406189555126 | 12.212765957446807 | 11.365570599613152 | 11.359767891682786 | 11.2321083172147 | 10.912959381044487 | 11.02321083172147 |
| 2.5 | 0.9690522243713733 | 12.216634429400385 | 10.743230174081237 | 0.9729206963249516 | 0.9303675048355898 | 0.9806576402321083 | 0.9032882011605415 | 12.313346228239846 | 12.007736943907156 | 12.456479690522244 | 11.79110251450677 | 11.317214700193421 | 11.042553191489361 | 10.41779497098646 | 11.317214700193421 |
| 5 | 0.9274661508704061 | 11.652321083172147 | 11.040135396518377 | 1.0270793036750483 | 1.0251450676982592 | 0.9806576402321083 | 0.9303675048355898 | 12.338491295938104 | 11.943907156673115 | 12.332688588007736 | 12.005802707930368 | 11.802707930367504 | 10.911025145067699 | 10.669245647969051 | 10.982591876208897 |
### Chart
| Category | | | | | | | | | | | | | | | |
|---|---|---|---|---|---|---|---|---|---|---|---|---|---|---|---|
| 0 | 1.0 | 13.737596899224805 | 13.403875968992248 | 0.9844961240310077 | 0.972093023255814 | 0.9271317829457364 | 0.9348837209302325 | 13.53798449612403 | 13.283720930232558 | 14.023255813953488 | 13.102325581395348 | 13.351937984496123 | 12.29767441860465 | 13.044961240310078 | 12.168992248062017 |
| 1 | 0.9883720930232558 | 13.776744186046512 | 12.81782945736434 | 0.9674418604651162 | 1.0046511627906975 | 1.0077519379844961 | 0.9069767441860466 | 13.51937984496124 | 14.347286821705426 | 14.73953488372093 | 13.23875968992248 | 13.862015503875968 | 13.510077519379843 | 12.399999999999999 | 12.558139534883722 |
| 2.5 | 0.9810077519379845 | 14.155038759689923 | 12.836434108527133 | 1.0248062015503876 | 0.9705426356589147 | 0.9937984496124032 | 0.9519379844961241 | 14.22170542635659 | 14.062015503875969 | 13.927131782945736 | 13.634108527131781 | 13.34108527131783 | 12.603100775193797 | 12.896124031007751 | 13.744186046511627 |
| 5 | 0.9558139534883721 | 13.477131782945737 | 12.881007751937986 | 1.0232558139534884 | 1.006201550387597 | 0.9953488372093022 | 1.0294573643410851 | 13.671317829457365 | 13.413953488372092 | 13.930232558139535 | 13.933333333333334 | 13.06046511627907 | 12.86046511627907 | 13.004651162790697 | 13.052713178294573 |
### Chart
| Category | | | | | | | | | | | | | | | |
|---|---|---|---|---|---|---|---|---|---|---|---|---|---|---|---|
| 0 | 1.0 | 10.394683026584868 | 4.459100204498977 | 0.8139059304703476 | 1.1247443762781186 | 1.1247443762781186 | 0.8507157464212679 | 10.27402862985685 | 6.883435582822086 | 6.629856850715746 | 3.7096114519427403 | 4.7607361963190185 | 5.10838445807771 | 4.969325153374233 | 4.16359918200409 |
| 1 | 0.9826175869120655 | 7.097137014314929 | 4.833333333333334 | 0.8629856850715747 | 1.0102249488752557 | 1.0102249488752557 | 1.1574642126789365 | 10.057259713701432 | 6.134969325153374 | 6.76482617586912 | 4.76482617586912 | 4.355828220858895 | 4.605316973415133 | 4.081799591002045 | 2.9447852760736195 |
| 2.5 | 0.9826175869120655 | 6.653374233128834 | 4.4815950920245395 | 1.116564417177914 | 0.8957055214723927 | 0.8957055214723927 | 1.0920245398773005 | 10.83435582822086 | 8.130879345603272 | 6.9079754601226995 | 5.652351738241308 | 4.08997955010225 | 4.52760736196319 | 4.576687116564417 | 3.5337423312883436 |
| 5 | 0.9652351738241308 | 4.706543967280163 | 3.5224948875255624 | 1.2065439672801637 | 0.8997955010224948 | 0.8997955010224948 | 0.7607361963190185 | 10.413087934560327 | 7.2392638036809815 | 6.310838445807771 | 4.699386503067485 | 4.629856850715746 | 5.0920245398773005 | 4.298568507157464 | 3.4478527607361964 |cde
de
be
be
bd
b
bc
ef
f
ef
de
cd
bd
bc
b
e
d
NF-κB activity
(fold change)
IRF activity
(fold change)
NF-κB activity
(fold change)
d
c
c
bc
bc
b
a
a
a
a
a
a
a
a
a
a
a
a
ML385 (µM)
AEM1 (µM)
AEM1 (µM)
Supplementary Figure S3

## Slide 6
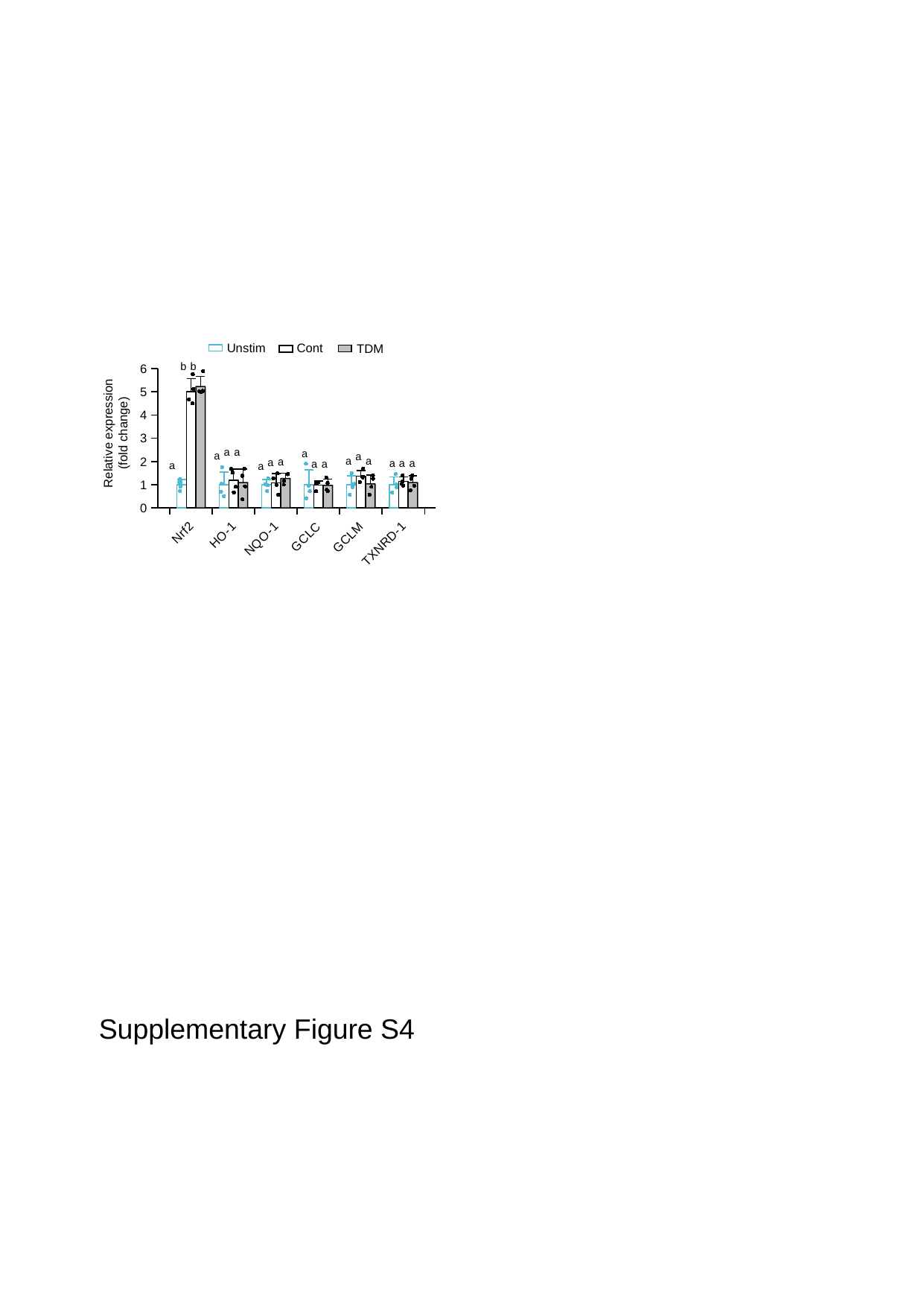

Unstim
Cont
TDM
b
b
[unsupported chart]
Relative expression
(fold change)
a
a
a
a
a
a
a
a
a
a
a
a
a
a
a
a
Supplementary Figure S4

## Slide 7
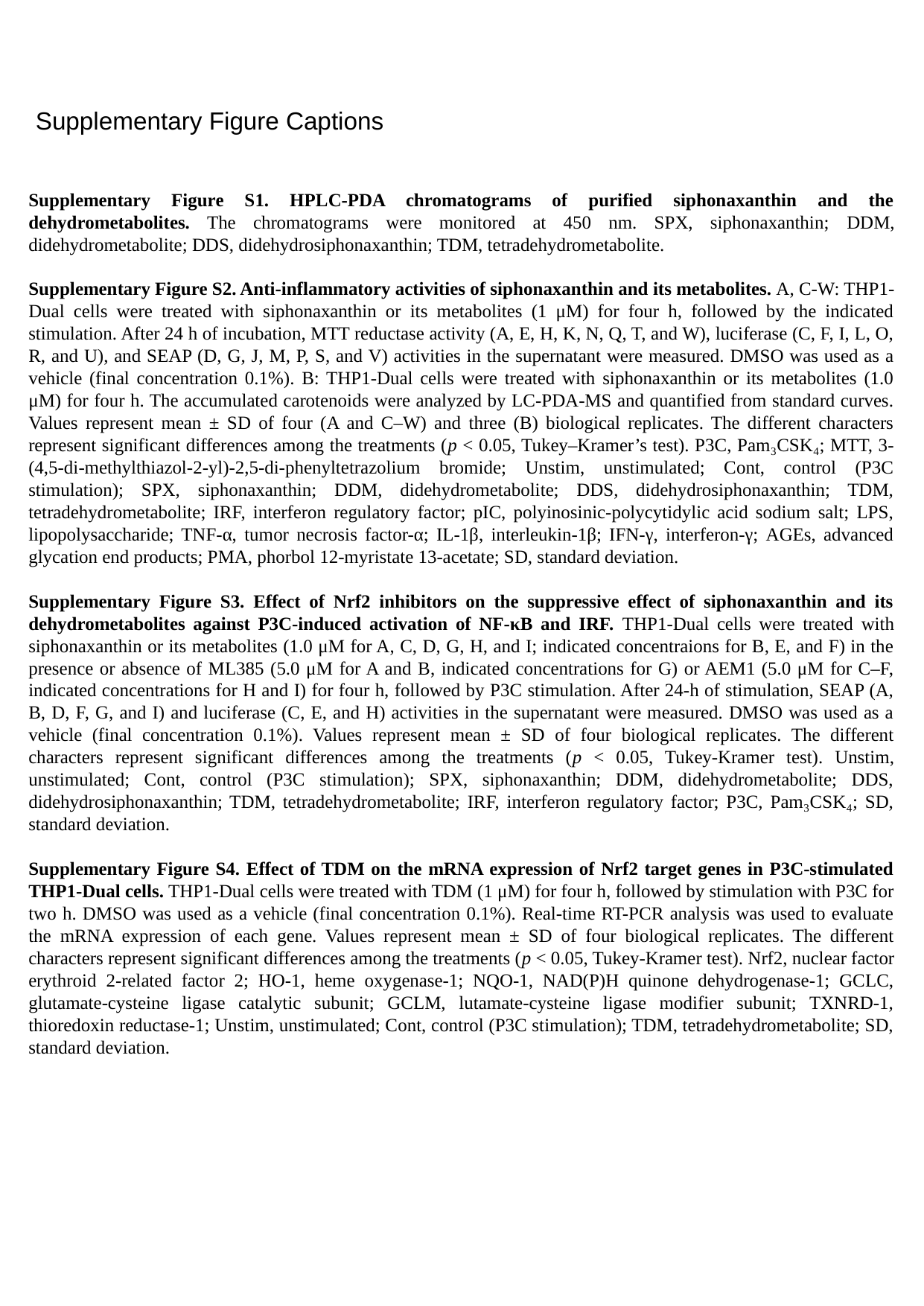

Supplementary Figure Captions
Supplementary Figure S1. HPLC-PDA chromatograms of purified siphonaxanthin and the dehydrometabolites. The chromatograms were monitored at 450 nm. SPX, siphonaxanthin; DDM, didehydrometabolite; DDS, didehydrosiphonaxanthin; TDM, tetradehydrometabolite.
Supplementary Figure S2. Anti-inflammatory activities of siphonaxanthin and its metabolites. A, C-W: THP1-Dual cells were treated with siphonaxanthin or its metabolites (1 μM) for four h, followed by the indicated stimulation. After 24 h of incubation, MTT reductase activity (A, E, H, K, N, Q, T, and W), luciferase (C, F, I, L, O, R, and U), and SEAP (D, G, J, M, P, S, and V) activities in the supernatant were measured. DMSO was used as a vehicle (final concentration 0.1%). B: THP1-Dual cells were treated with siphonaxanthin or its metabolites (1.0 μM) for four h. The accumulated carotenoids were analyzed by LC-PDA-MS and quantified from standard curves. Values represent mean ± SD of four (A and C–W) and three (B) biological replicates. The different characters represent significant differences among the treatments (p < 0.05, Tukey–Kramer’s test). P3C, Pam₃CSK₄; MTT, 3-(4,5-di-methylthiazol-2-yl)-2,5-di-phenyltetrazolium bromide; Unstim, unstimulated; Cont, control (P3C stimulation); SPX, siphonaxanthin; DDM, didehydrometabolite; DDS, didehydrosiphonaxanthin; TDM, tetradehydrometabolite; IRF, interferon regulatory factor; pIC, polyinosinic-polycytidylic acid sodium salt; LPS, lipopolysaccharide; TNF-α, tumor necrosis factor-α; IL-1β, interleukin-1β; IFN-γ, interferon-γ; AGEs, advanced glycation end products; PMA, phorbol 12-myristate 13-acetate; SD, standard deviation.
Supplementary Figure S3. Effect of Nrf2 inhibitors on the suppressive effect of siphonaxanthin and its dehydrometabolites against P3C-induced activation of NF-κB and IRF. THP1-Dual cells were treated with siphonaxanthin or its metabolites (1.0 μM for A, C, D, G, H, and I; indicated concentraions for B, E, and F) in the presence or absence of ML385 (5.0 μM for A and B, indicated concentrations for G) or AEM1 (5.0 μM for C–F, indicated concentrations for H and I) for four h, followed by P3C stimulation. After 24-h of stimulation, SEAP (A, B, D, F, G, and I) and luciferase (C, E, and H) activities in the supernatant were measured. DMSO was used as a vehicle (final concentration 0.1%). Values represent mean ± SD of four biological replicates. The different characters represent significant differences among the treatments (p < 0.05, Tukey-Kramer test). Unstim, unstimulated; Cont, control (P3C stimulation); SPX, siphonaxanthin; DDM, didehydrometabolite; DDS, didehydrosiphonaxanthin; TDM, tetradehydrometabolite; IRF, interferon regulatory factor; P3C, Pam₃CSK₄; SD, standard deviation.
Supplementary Figure S4. Effect of TDM on the mRNA expression of Nrf2 target genes in P3C-stimulated THP1-Dual cells. THP1-Dual cells were treated with TDM (1 μM) for four h, followed by stimulation with P3C for two h. DMSO was used as a vehicle (final concentration 0.1%). Real-time RT-PCR analysis was used to evaluate the mRNA expression of each gene. Values represent mean ± SD of four biological replicates. The different characters represent significant differences among the treatments (p < 0.05, Tukey-Kramer test). Nrf2, nuclear factor erythroid 2-related factor 2; HO-1, heme oxygenase-1; NQO-1, NAD(P)H quinone dehydrogenase-1; GCLC, glutamate-cysteine ligase catalytic subunit; GCLM, lutamate-cysteine ligase modifier subunit; TXNRD-1, thioredoxin reductase-1; Unstim, unstimulated; Cont, control (P3C stimulation); TDM, tetradehydrometabolite; SD, standard deviation.
